# Effects of dienogest treatment on endometrioma-related clinical symptoms and endometrioma size: retrospective cohort study

**DOI:** 10.3389/fmed.2025.1581661

**Published:** 2025-05-01

**Authors:** Ufuk Atlihan, Onur Yavuz, Can Ata, Huseyin Aytug Avsar, Selcuk Erkilinc

**Affiliations:** ^1^Department of Obstetrics and Gynecology, Merkezefendi Hospital, Manisa, Türkiye; ^2^Department of Obstetrics and Gynecology, Dokuz Eylul University School of Medicine, Izmir, Türkiye; ^3^Department of Obstetrics and Gynecology, Buca Seyfi Demirsoy Training and Research Hospital, Izmir Democracy University, Izmir, Türkiye; ^4^Department of Obstetrics and Gynecology, Tinaztepe University School of Medicine Galen Hospital, Izmir, Türkiye

**Keywords:** cyclic pelvic pain, dysmenorrhea, dyspareunia, endometrioma, dienogest

## Abstract

**Background:**

To evaluate the efficacy and long-term safety of treatment with dienogest in patients with endometrioma.

**Methods:**

Patients with endometrioma-related chronic pelvic pain were included in this retrospective study from March 2018 to March 2023. Enrolled patients received 2 mg of dienogest once daily. Data from 180 patients were analyzed. Group 2 (*n* = 104, 57.8%), comprising patients undergoing long-term therapy (>12 months), was compared with group 1 (*n* = 76, 42.2%), consisting of patients undergoing short-term therapy (<2 months), regarding their response to changes in endometrioma size and visual analog scale (VAS) scores. Statistical analysis was performed using the SPSS version 26.0 software. Non-normally distributed parameters were analyzed using the Mann–Whitney U test. In the evaluation of the data, apart from identifying statistical methods, the t-test was used in comparison of paired groups, and the matched t-test was used in the determination of changes before and after treatment. The Chi-square test and Fisher’s precision test were used in the analysis of categorical data. Categorical variables are presents as percentages, and quantitative variables are summarized as mean (95% confidence intervals) and median (minimum-maximum). *p*-values of <0.05 were considered statistically significant.

**Results:**

Findings at T0 (baseline) and T1 (sixth month) visits, in which the entire study cohort could be included, were compared. Then, patients who continued treatment at visits every 6 months after T1 (>12 months) were compared one by one with the findings at T0. The reduced libido was 4.3 times higher in the long-term group, but the weight gain was higher in the short-term group. Analysis within all patients and individual groups (short term vs. long term) showed a significant decrease in endometrioma size and VAS scores between T0 and T1 visit findings. Similarly, the findings of T2 and each subsequent visit of the patients in the long-term group were compared with the initial findings and a significant reduction in endometrioma size and VAS scores was observed.

**Conclusion:**

Although the effectiveness of dienogest treatment for endometrioma seems to begin in the sixth month, its effectiveness maximizes in patients whose treatment duration is over 1 year.

## 1 Background

Endometriosis is a chronic estrogen-dependent disease characterized by the presence of endometrial glands and stroma outside the uterus (1). It affects approximately 10–15% of women of reproductive age (2). Endometriomas are present in up to 41% of patients with endometriosis (3, 4). The most common symptoms of these patients are pelvic pain and infertility (5). The definitive diagnosis of endometriosis is the histopathologic evaluation of samples taken during laparoscopy surgeries. The diagnosis of endometriosis can only be suspected based on medical history, clinical symptoms, physical examination, and imaging methods (6). Medical treatment, surgery, assisted reproductive techniques for infertility, and combination of these treatments are options for the management of endometriosis (4, 7, 8). Hormonal therapy is widely used as first-line treatment in symptomatic women and in women for whom surgery is not recommended (8). Commonly used hormonal treatments include gonadotropin-releasing hormone agonist (GnRHa), combined oral contraceptives, and progestins. The goal of these treatments is to reduce the size of endometriotic foci and associated symptoms (6).

Progestins are a suitable option to prevent the proliferation of estrogen-induced lesions and reduce the pain associated with endometriosis. Dienogest, one of the progestins approved for the treatment of endometriosis, has a strong progestogenic effect, moderate estrogen suppressing effect, anti-inflammatory, antiproliferative, and antiangiogenic properties. These properties effectively reduce the growth of endometriosis lesions (9–12). Studies have shown that dienogest, administered at a daily dose of 2 mg, reduces pain in endometriosis at a significantly higher rate than placebo. In addition, it has been shown that the level of pain relief is similar to GnRHa by providing less hypoestrogenemia than GnRHa. Moreover, dienogest has been reported to be more effective than norethindrone acetate, another progestin used in the treatment of endometriosis, and has a lower risk of adverse effects (13–16).

The results of the few studies in the literature investigating dienogest treatment for endometrioma vary in terms of endometrioma size, clinical symptoms, and adverse effects. Endometriosis tends to recur after surgery in as many as 89.6% of cases. Endometrioma recurrence may be prevented with postoperative long-term (>12 months) hormonal treatment (8). Maiorana et al. reported that long-term (>15 months) dienogest treatment for endometrioma significantly reduced the clinical symptoms of patients, but did not have a similar effect on endometrioma sizes (2). Gokmen et al. revealed that dienogest treatment for endometrioma treatment significantly reduced both clinical symptoms and endometrioma size at the end of 6 months compared with baseline (17). The duration of treatment to significantly reduce pelvic pain in women treated with dienogest after endometriosis surgery varies across studies (18–20).

This study describes our center’s experience with dienogest (2 mg) throughout treatment periods in women with endometriomas and also compares clinical symptoms, endometrioma sizes, and adverse effect findings in a group of patients who received long-term (>12 months) treatment with those who received short-term (<12 months) treatment.

## 2 Materials and methods

This was a retrospective cohort study conducted at Dokuz Eylul Hospital. Informed consent was obtained from all participants included in this study. The study was performed in line with principles of the Declaration of Helsinki. Institutional ethics committee approval was provided (Date: 31/01/2024, File Number: 8659-GOA).

**Figure 1:**
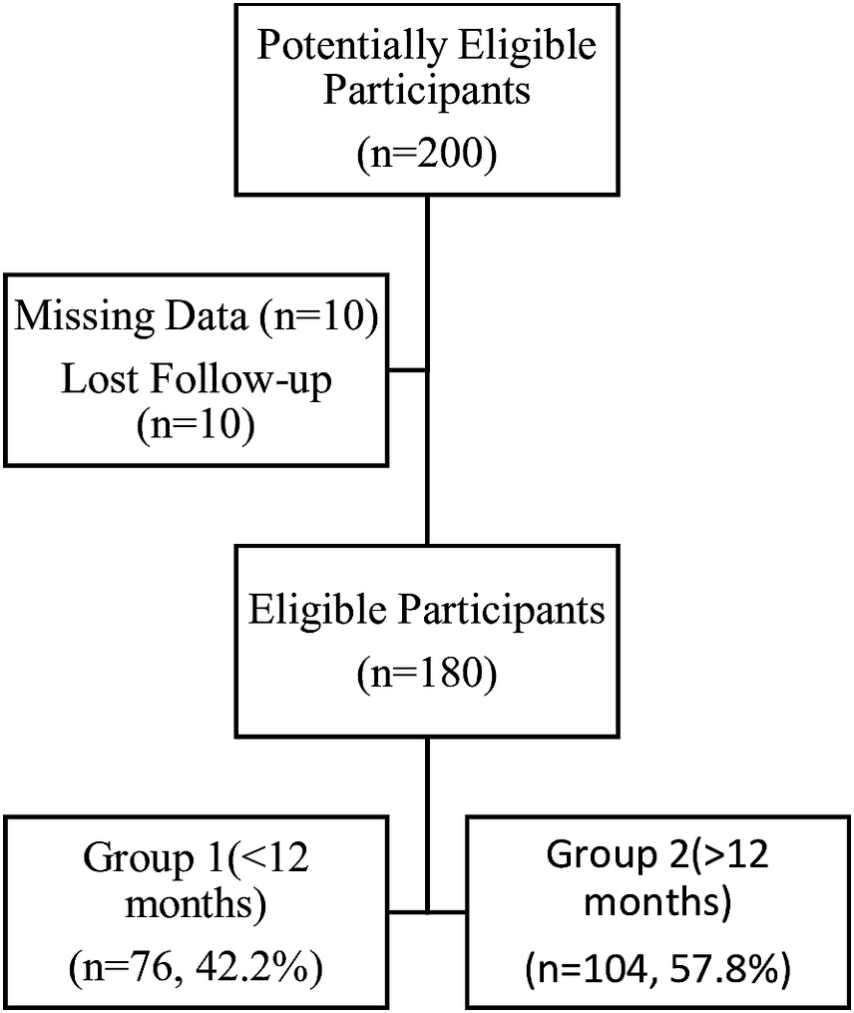
Flow chart of the study.

Patients aged over 18 years who had a clinical diagnosis of endometriosis, a clinical indication for medical therapy, and no need for contraception were included in the study. A history of endometriosis surgery more than 24 months ago, any history of medical treatment for endometriosis, pregnancy status, seeking pregnancy, suspicion of neoplasm, and contraindications to progestin therapy were exclusion criteria for the study. We evaluated 200 patients who were treated and followed up in Dokuz Eylul Hospital between March 2018 and March 2023, had a clinical diagnosis of unilateral or bilateral endometrioma, experienced chronic pelvic pain, and were therefore using 2 mg of dienogest daily. Records were missing for 10 patients, and 10 patients were lost to follow-up. The remaining 180 patients were included in the study analysis. Group 2 (*n* = 104, 57.8%), comprising patients undergoing long-term therapy (>12 months), was compared with group 1 (*n* = 76, 42.2%), consisting of patients undergoing short-term therapy (<12 months), regarding their response to changes in endometrioma size and visual analog scale (VAS) scores (Figure 1).

Patients’ demographic characteristics were recorded before treatment. During the initial visit, each patient underwent a thorough gynecologic examination using transvaginal ultrasound. Ovarian endometrioma was diagnosed using transvaginal ultrasound performed by an experienced sonographer (UA) utilizing ‘pattern recognition’ through subjective evaluation of grayscale and Doppler-ultrasound characteristics. The diagnosis of endometrioma was made according to the International Ovarian Tumor Analysis (IOTA) rules when ‘an adnexal mass with ground-glass echogenicity of the cyst fluid, one to four locules, and no papillations with detectable blood flow’ was observed (21). Endometrioma diameters were measured by taking the mean of the largest horizontal and vertical components. The endometrioma size was recorded in millimeters. Bilateral endometriomas were measured separately, and their mean size was recorded. Symptoms of dysmenorrhea, dyspareunia, and nonmenstrual pelvic pain were evaluated using VAS scores ranging from 0 to 10, where 0 indicates no pain and 10 indicates severe pain (22). The second follow-up visit was conducted at 6 months to assess changes in the patients’ pain symptoms and endometrioma sizes. These visits were repeated every 6 months. Endometrioma sizes and VAS scores were evaluated at each visit. The VAS scores and endometrioma sizes were recorded at baseline (T0) and subsequently at 6 months (T1), 12 months (T2), 18 months (T3), 24 months (T4), and 30 months (T5). Additionally, the occurrence of adverse effects such as spotting, headache, reduced libido, and weight gain were assessed. The presence of these symptoms at any visit during the treatment was recorded as an adverse effect.

Statistical analysis was performed using the SPSS version 26.0 software (IBM Inc., Chicago, IL, United States). The normality of the distribution was evaluated using the Kolmogorov–Smirnov test. Non-normally distributed parameters were analyzed using the Mann–Whitney U test. In the evaluation of the data, apart from the identifying statistical methods, the t-test was used in paired group comparisons, and the matched t-test was used in the determination of changes before and after treatment. The Chi-square test and Fisher’s precision test were used in the analysis of categorical data. Regarding the statistical study categorical variables as percentage, quantitative variables were summarized as mean (95% confidence intervals) and median (minimum-maximum). *p*-values of <0.05 were considered statistically significant.

## 3 Results

In this study, the results of 180 patients were analyzed. Group 2 (*n* = 104, 57.8%), comprising patients undergoing long-term therapy (>12 months), was compared with group 1 (*n* = 76, 42.2%), consisting of patients undergoing short-term therapy (<12 months). Patients were visited every 6 months.

There were 180 patients at the baseline (T0) and 6th month (T1) visits. There were 104 (57.8%) patients at the 12th month visit, 65 (36.1%) at the 18th month visit, 17 (9.4%) at the 24th month visit, and four (2.2%) at the 30th month visit.

The comparison of the group characteristics are listed in Table 1. The age of all patients was between 29 and 40 (median: 34) years. The median age of group 2 was older than group 1 (*p* < 0.001). The body mass index was lower in group 1 (22 vs. 23, *p* < 0.001). The groups were similar in terms of menarche age, nulliparity, pelvic surgery history, endometriosis surgery history, and number of pelvic surgeries. The treatment follow-up period for all patients was between 6 and 32 (median: 16) months. The treatment follow-up period of group 1 was 9 (6–11) months and group 2 was 19 (14–32) months.

**Table 1:** Comparison of characteristics of groups.

Variables	All patients (*n* = 180, 100%)	Group 1 (*n* = 76, 42.2%)	Group 2 (*n* = 104, 57.8%)	*p*-value
Age (years)	34 (29–40)	33 (29–37)	35 (31–40)	<0.001
Menarche age (years)	12 (10–13)	12 (11–13)	12 (10–13)	0.7
Body mass index (kg/m^2^)	23 (19–29)	22 (19–25)	23 (20–29)	<0.001
Nulliparity (n, %)	89 (49.4%)	34 (44.7%)	55 (52.9%)	0.2
Pelvic surgery history (n, %)	27 (15%)	11 (14.5%)	16 (15.4%)	0.8
Endometriosis surgery history (n, %)	10 (5.6%)	2 (2.6%)	8 (7.7%)	0.1
Number of pelvic surgeries (n)	0 (0–3)	0 (0–2)	0 (0–3)	0.7
Treatment follow-up period (months)	16 (6–32)	9 (6–11)	19 (14–32)	<0.001

The comparison of drug-related adverse effects of the groups is shown in Table 2. Reduced libido was higher in group 2, and weight gain was higher in group 1 (10.5% vs. 45.2%, *p* < 0.001, 88.2% vs. 76%, *p* = 0.02, respectively). Spotting and headache were similar between the groups (*p* = 0.3 and *p* = 0.07, respectively).

**Table 2:** Comparison of drug-related side effects of groups.

Variables	All patients (*n* = 180, 100%)	Group 1 (*n* = 76, 42.2%)	Group 2 (*n* = 104, 57.8%)	*p*-value
Spotting (n, %)	44 (24.4%)	16 (21.1%)	28 (26.9%)	0.3
Headache (n, %)	32 (17.8%)	9 (11.8%)	23 (22.1%)	0.07
Reduced libido (n, %)	55 (30.6%)	8 (10.5%)	47 (45.2%)	<0.001
Weight gain (n, %)	146 (81.1%)	67 (88.2%)	79 (76%)	0.02

Table 3 shows the analysis of mean change in endometrioma size and VAS from baseline to T1 by groups. The location of endometrioma was statistically significantly different between the groups (*p* < 0.001). The rate of right-located endometrioma was higher in group 1, and bilateral endometrioma was higher in group 2 (52.6% vs. 29.8%, *p* = 0.002, 13.2% vs. 39.8%, *p* < 0.001, respectively).

**Table 3:** Analysis of mean change in endometrioma size and VAS from baseline to T1 by groups.

Variables	All patients (*n* = 180, 100%)	Group 1 (*n* = 76, 42.2%)	Group 2 (*n* = 104, 57.8%)	*p* value*
Location of endometrioma (n, %)				<0.001
Right	71 (39.4%)	40 (52.6%)	31 (29.8%)	0.002
Left	58 (32.3%)	26 (34.2%)	32 (30.8%)	0.6
Bilateral	51 (28.3%)	10 (13.2%)	41 (39.8%)	<0.001
Endometrioma size (mm) at T0	33 (28–39)	33 (28–29)	33 (28–39)	0.8
Endometrioma size (mm) at T1	30 (24–37)	29.5 (24–37)	30 (25–37)	0.9
*p*-value**	−3.1 (−3.2 to −2.9) <0.001	−3.1 (−3.4 to −2.9) <0.001	−3.1 (−3.2 to −2.9) <0.001	
Variation from T0 to T1 of size the endometrioma (mm)	−3 (−6 to 0)	−3 (−6 to 0)	−3 (−6 to −2)	0.8
Dysmenorrhea VAS at T0	8 (6–9)	7 (6–9)	8 (6–9)	0.002
Dysmenorrhea VAS at T1	6 (4–8)	6 (4–7)	7 (5–8)	<0.001
*p*-value**	−1.5 (−1.1 to −0.9) <0.001	−1.7 (−1.9 to −1.5) <0.001	−1.5 (−1.6 to −1.3) <0.001	
Variation from T0 to T1 of dysmenorrhea VAS	−1 (−4 to 0)	−2 (−4 to 0)	−1 (−3 to 0)	0.003
Dysparonia VAS at T0	6 (3–8)	5 (3–7)	6 (4–8)	0.007
Dysparonia VAS at T1	5 (2–7)	4 (2–6)	5 (3–7)	<0.001
*p* value**	−0.6 (−0.7 to −0.5) <0.001	−0.8 (−0.9 to −0.7) <0.001	−0.6 (−0.7 to −0.5) <0.001	
Variation from T0 to T1 of dysparonia VAS	−1 (−2 to 0)	−1 (−2 to 0)	−1 (−1 to 0)	0.001
Nonmenstrual pelvic pain VAS at T0	6 (4–8)	6 (4–7)	6 (5–8)	<0.001
Nonmenstrual pelvic pain VAS at T1	5 (3–6)	4 (3–6)	5 (3–6)	<0.001
*p* value**	−1 (−1.1 to −0.9) <0.001	−1.1 (−1.2 to −0.9) <0.001	−1 (−1.1 to −0.9) <0.001	
Variation from T0 to T1 of nonmenstrual pelvic pain VAS	−1 (−4 to 1)	−1 (−4 to 1)	−1 (−3 to 0)	0.5

VAS, Visual analog scale of pain. p^*^ value: Mann–Whitney U test; p^**^ value: paired *t*-test.

Dysmenorrhea VAS scores at both T0 and T1 were higher in group 2 (7 vs. 8, *p* = 0.02, 6 vs. 7, *p* < 0.001, respectively). In both groups, the dysmenorrhea VAS scores at T1 decreased statistically significantly compared with T0 (*p* < 0.001). The variation in dysmenorrhea VAS scores from T0 to T1 differed significantly between the groups (*p* = 0.003).

Dyspareunia VAS scores at both T0 and T1 were also higher in group 2 (5 vs. 6, *p* = 0.007, 4 vs. 5, *p* < 0.001, respectively). In both groups, dyspareunia VAS scores at T1 decreased significantly compared with T0 (*p* < 0.001). However, the reduction in dyspareunia VAS scores from T0 to T1 was statistically greater in group 1 (*p* = 0.001).

Non-menstrual pelvic pain VAS scores at both T0 and T1 were higher in group 2 (*p* < 0.001). Similarly, in both groups, the nonmenstrual pelvic pain VAS scores at T1 decreased significantly compared with T0 (*p* < 0.001). However, the variation in nonmenstrual pelvic pain VAS scores from T0 to T1 did not differ significantly between the groups (*p* = 0.5).

The analysis of the mean change in endometrioma size from baseline at various time intervals is presented in Table 4.

**Table 4:** Analysis of mean change in endometrioma size from baseline by time intervals.

Variables (n, %)	Results*
Endometrioma size (mm) at T0 (*n* = 180,100%)	33 (32.5–33.4)
Endometrioma size (mm) at T1 (*n* = 180,100%)	29.8 (29.4–30.3)
*p*-value**	−3.1 (−3.2 to −2.9) <0.001
Endometrioma size (mm) at T0 (*n* = 104, 57.7%)	32.9 (32.4–33.4)
Endometrioma size (mm) at T2 (*n* = 104, 57.7%)	28.5 (28–29)
*p*-value**	−4.4 (−4.5 to −4.2) <0.001
Endometrioma size (mm) at T0 (*n* = 65, 36.1%)	32.8 (32.2–33.5)
Endometrioma size (mm) at T3 (*n* = 65, 36.1%)	27.1 (26.5–27.8)
*p*-value**	−5.7 (−6 to −5.3) <0.001
Endometrioma size (mm) at T0 (*n* = 17, 9.4%)	32.3 (31–33.7)
Endometrioma size (mm) at T4 (*n* = 17, 9.4%)	25.2 (24–26.3)
*p*-value**	−7.1 (−8 to −6.1) <0.001
Endometrioma size (mm) at T0 (*n* = 4, 2.2%)	32 (26.9–37)
Endometrioma size (mm) at T5 (*n* = 4, 2.2%)	23.5 (18.5–28.4)
*p*-value**	−8.5 (−10.5 to −6.4) 0.001

VAS, Visual analog scale of pain. p^*^ value: Mann–Whitney U test; p^**^ value: paired *t*-test.

The analysis of mean change in dysmenorrhea, dysparonia, and nonmenstrual pelvic pain VAS scores from baseline by time intervals is presented in Table 5. Endometrioma sizes, dysmenorrhea, dysparonia, and nonmenstrual pelvic pain VAS scores at all visits from T1 to T5 were compared with T0, and each comparison was statistically significant.

**Table 5:** Analysis of mean change in dysmenorrhea, dysparonia, and nonmenstrual pelvic pain visual analog scale scores from baseline by time intervals.

Variables (n, %)	Dysmenorrhea	Dysparonia	Nonmenstrual pelvic pain
VAS at T0 (*n* = 180, 100%)	7.5 (7.4–7.6)	5.5 (5.4–5.6)	6 (5.8–6.1)
VAS at T1 (*n* = 180, 100%)	6 (5.9–6.1)	4.8 (4.7–4.9)	4.9 (4.8–5)
*p*-value*	−1.5 (−1.6 to −1.3) <0.001	−0.6 (−0.7 to −0.5) <0.001	−1 (−1.1 to −0.9) <0.001
VAS at T0 (*n* = 104, 57.8%)	7.7 (7.5–7.9)	5.7 (5.5–5.8)	6.3 (6.1–6.4)
VAS at T2 (*n* = 104, 57.8%)	5.3 (5.2–5.5)	4.1 (4–4.3)	3.6 (3.5–3.7)
*p*-value*	−2.3 (−2.5 to −2.2) <0.001	−1.5 (−1.6 to −1.3) <0.001	−2.6 (−2.8 to −2.5) <0.001
VAS at T0 (*n* = 65, 36.1%)	7.7 (7.5–7.9)	5.6 (5.4–5.9)	6.3 (6.1–6.5)
VAS at T3 (*n* = 65, 36.1%)	4.4 (4.2–4.6)	3.3 (3.2–3.5)	2.6 (2.5–2.8)
*p*-value*	−3.2 (−3.5 to −3) <0.001	−2.3 (−2.5 to −2.1) <0.001	−3.7 (−3.9 to −3.4) <0.001
VAS at T0 (*n* = 17, 9.4%)	8 (7.7–8.3)	5.7 (5.4–6.1)	6.5 (6.2–6.9)
VAS at T4 (*n* = 17, 9.4%)	4.2 (3.9–4.5)	3.1 (2.8–3.3)	2.8 (2.7–3)
*p-*value*	−3.8 (−4.2 to −3.4) <0.001	−2.6 (−3 to −2.2) <0.001	−3.7 (−4.1 to −3.7) <0.001
VAS at T0 (*n* = 4, 2.2%)	8.2 (6.7–9.7)	8.2 (7.3–9.1)	7 (7–7)
VAS at T5 (*n* = 4, 2.2%)	2.7 (1.2–4.2)	2.7 (1.9–3.5)	2 (0.7–3.2)
*p*-value*	−5.5 (−8.5 to −2.4) <0.001	−3.2 (−4 to −2.4) 0.001	−5 (−6.2 to −3.7) 0.001

VAS, Visual analog scale of pain. p^*^ value: Mann–Whitney U test; p^**^ value: paired *t*-test.

## 4 Discussion

In this current study, we evaluated endometrioma size, clinical symptoms, and the adverse effects of short-term (<12 months) and long-term (>12 months) dienogest treatment for endometrioma. Findings at T0 (baseline) and T1 (sixth month) visits, in which the entire study cohort could be included, were compared. Then, patients who continued treatment at visits every 6 months after T1 (>12 months) were compared one by one with the findings at T0. The reduced libido was 4.3 times higher in the long-term group, but the weight gain was higher in the short-term group. Analysis within all patients and individual groups (short-term vs. long-term) showed a significant decrease in endometrioma size and clinical symptoms between T0 and T1 visit findings. Similarly, the findings of T2 and each subsequent visit of the patients in the long-term group were compared with the initial findings. A significant reduction in endometrioma size and clinical symptoms was observed.

Many physicians indicate that medical management of endometriosis should be empirical before laparoscopic confirmation of the disease (23). Surgery is invasive, expensive, and carries a risk of morbidity compared with medical treatment (24). Therefore, the indications and risks of surgery should be explained and discussed in detail to the patient. First-line treatment includes analgesic and anti-inflammatory medications, combined birth control pills, and progestins such as medroxyprogesterone acetate or norethisterone (24–26). Progestins and estrogen-progestogen combinations have repeatedly been shown to be safe, well-tolerated, inexpensive, and effective in the long-term treatment of women with symptomatic endometriosis (27).

GnRH analogs are an effective treatment for endometriosis but are associated with symptoms of hypoestrogenism. Long-term use reduces bone mineral density (28). Dienogest is a fourth-generation selective progestin that binds more specifically to progesterone receptors, has a local effect on endometriotic lesions, has little androgenic, estrogenic, glucocorticoid or mineralocorticoid activity, and has a minimal effect on metabolic parameters (29). In a meta-analysis, drug-related adverse effects were compared between dienogest and GnRHa treatments. Although a higher rate of spotting and weight gain adverse effects were detected in the dienogest group, hot flashes and vaginal dryness tended to be lower (30). Conversely, it was reported that dienogest maintenance treatment after surgery caused fewer general adverse effects, especially spotting and weight gain, compared with other medical treatments in another meta-anlysis (31). Maiorana et al. examined the effectiveness of dienogest treatment for endometrioma treatment in two groups as shorter and longer than 15 months (2). Among all patients, reduced libido was the most common adverse effect (32.7%), followed by osteopenia (27.6%), spotting (22.9%), weight gain (17.8%), and headache (17.2%). Reduced libido, weight gain, and headache were more frequent in the long-term group. In a review by Andreas et al., dienogest treatment saw less reduction in bone mass compared with leuprolide acetate and intranasal buserelin (23). In our study, unfortunately, we could not evaluate osteopenia. Weight gain was found to be the most common adverse effect, followed by reduced libido. Reduced libido was higher in the long-term group, and weight gain was higher in the short-term group. Additionally, Andreas et al. reported that most of the patients evaluated in studies using dienogest had spotting. However, in patients who were able to continue treatment for a long time, the frequency and intensity of bleeding tended to decrease over the determined treatment period (23). Although spotting symptoms were detected more frequently in the long-term group in our study, the difference between the groups was not statistically significant.

Torre et al. reported a statistically significant reduction in clinical symptoms associated with chronic pelvic pain in both endometrioma and other endometriosis phenotype groups receiving at least 12 months of dienogest treatment (32). Another study found a significant reduction in major endometriosis-related symptoms such as dysmenorrhea, dyspareunia, and nonmenstrual pelvic pain. At the end of the first year, there was a clear decrease in dysmenorrhea. Other symptoms gradually decreased over a longer period. Although endometrioma showed a decrease compared with baseline at the end of 1 year, this decrease was not statistically significant (2). In the study by Gokmen et al., dysmenorrhea and dyspareunia symptoms, along with endometrioma sizes, showed a significant decrease in the third and sixth months compared with the initial findings (17). In a systematic review evaluating dienogest as a maintenance treatment for endometriosis after surgery, a statistically significant decrease was found in the clinical symptom VAS scores of the treatment group compared with the untreated group at the third month (31). A statistically significant decrease was detected in the clinical symptom VAS scores of the group receiving dienogest treatment compared with the group receiving other treatments in the twelfth month (31). Similarly, two other studies found that dienogest treatment resulted in improvement in VAS scores of clinical symptoms with tolerable adverse effects after 1 year (33, 34). According to our study, endometrioma size showed a significant decrease in both groups at the sixth month (T1). However, the variation of the sizes of the endometriomas was similar between groups. Although the rate of bilateral endometriomas was higher in the long-term group and endometrioma sizes were similar at the first (T0) and second (T1) visits of both groups, noticeably during this period, the variation of dysmenorrhea and dyspareunia VAS scores decreased statistically more in the short-term group. The variation of nonmenstrual pelvic pain VAS scores was similar between the groups. Moreover, endometrioma sizes, dysmenorrhea, dysparonia, and nonmenstrual pelvic pain VAS scores at all visits from T2 to T5 were compared with T0, and each comparison was statistically significant.

Our study evaluated the efficacy of dienogest during the follow-up period and did not compare its treatment efficacy with other treatment modalities, primarily combined oral contraceptives (COCs). There are studies on this subject in literature. In the study conducted by Piacenti et al., in a comparison of dienogest and continuous oral levonorgestrel/EE in patients with endometriosis, both treatments were effective and safe for patients with endometriosis (35). Patient compliance and adverse effects were similar in both groups, but women receiving dienogest had significantly greater reductions in endometriotic lesions, pain symptoms, and quality of life than women receiving continuous COC (35). In the study conducted by El Taha et al., dienogest was found to be comparable to COC in terms of relieving endometriosis-related pelvic pain and quality of life (36).

There are very few studies in literature regarding the effectiveness of dienogest. The limitations of the study were the small sample size for the maximum observation period and its retrospective design. Because we evaluated the patients with a non-invasive method, we could not perform surgical endometriosis staging. The strengths of our study are that it represents a comprehensive evaluation with a quality methodologic evaluation and strict inclusion criteria.

## 5 Conclusion

Although the effectiveness of dienogest treatment for endometrioma seems to begin in the sixth month, its effectiveness maximizes in patients whose treatment duration is over 1 year. Further comparative studies on long-term effectiveness and safety between patients with and without long-term medical treatment will be needed. Endometrioma treatment management by gynecologists in patients with infertility and suspected malignancy should be individualized. Future similar studies should focus on endometriosis phenotypes.

## Data Availability

The original contributions presented in the study are included in the article/supplementary material, further inquiries can be directed to the corresponding author.
